# Fractal Dimension Analysis of Texture Formation of Whey Protein-Based Foods

**DOI:** 10.1155/2018/7673259

**Published:** 2018-05-21

**Authors:** Robi Andoyo, Vania Dianti Lestari, Efri Mardawati, Bambang Nurhadi

**Affiliations:** Department of Food Industrial Technology, Faculty of Agro-Industrial Technology, Universitas Padjadjaran, Jl. Raya Bandung Sumedang Km. 21, Jatinangor, Sumedang 40600, Indonesia

## Abstract

Whey protein in the form of isolate or concentrate is widely used in food industries due to its functionality to form gel under certain condition and its nutritive value. Controlling or manipulating the formation of gel aggregates is used often to evaluate food texture. Many researchers made use of fractal analysis that provides the quantitative data (i.e., fractal dimension) for fundamentally and rationally analyzing and designing whey protein-based food texture. This quantitative analysis is also done to better understand how the texture of whey protein-based food is formed. Two methods for fractal analysis were discussed in this review: image analysis (microscopy) and rheology. These methods, however, have several limitations which greatly affect the accuracy of both fractal dimension values and types of aggregation obtained. This review therefore also discussed problem encountered and ways to reduce the potential errors during fractal analysis of each method.

## 1. Introduction

The network system of proteins is often applied to food, one of which is whey protein. This is because whey may affect the structure or texture as well as viscosity of foods depending on the processing applied [1]. Whey protein is also a high-quality protein that provides essential amino acids and has a high bioavailability (protein efficiency ratio or PER) compared to the other sources of protein [2]. This protein functionality is influenced by the ability of whey protein to form an aggregated network that can act as gelling agent in protein-fortified food. The textural properties of gel-type foods are principally determined by the combination of structural properties of the gel matrix and filler particles. The filler particles can be classified as “active” or “inactive” based on the resulting effects of these particles in rheological properties of gel. The “inactive” filler has a low chemical affinity on the polymer matrix so it cannot strengthen the resulting gel. The “active” filler has a strong interaction with the polymer matrix and therefore can strengthen the resulting gel structure [3]. It is important to predict or manipulate the ability of whey protein in forming gel in order to produce foods with desirable texture. Gelling properties of whey protein are necessary in determining consumer acceptance in many kinds of food products, such as processed meat, milk, bread, and cakes, and improving product appearance by preventing surface moisture in yogurt [1, 4].

The application of whey protein as both structure builder and structure breaker in food should therefore be conducted by manipulating the aggregation process of whey-based particles network to produce food products with controlled texture. This accounts for a fundamental and quantitative understanding of the aggregation process. Many studies had observed the kinetics and aggregation process of particle network dispersions both theoretically and experimentally. Whey protein can theoretically form or alter the food texture because it can undergo conformational changes and form network or gel aggregate under certain and continuous treatment. Whey protein aggregation process consists of three stages that often occur subsequently, including conformational changes, chemical reactions, and physical interactions [5], and can be done through heat or cold gelation methods. The rheological properties of protein gels produced from the process vary greatly, depending on pH, ionic strength, temperature and heating rate, and gelation methods used [6, 7].

Whey protein gels under certain length scales were proven to have self-similar structure; this structure can be described and quantified experimentally by fractal concept. The fractal concept can be used to describe and quantify the structure of aggregated particles by using fractal dimension *D* or *D*_*f*_—this shows the relationship between the number of particles on aggregate and their size, *N* ~ *R*^*D*^. *D*_*f*_ values of 1.7–1.8 represent diffusion-limited cluster-cluster aggregation (DLCA) while *D*_*f*_ value of 2.0–2.2 represents reaction-limited cluster-cluster aggregation (RLCA)—the higher *D*_*f*_ value also indicates a denser aggregate structure while lower *D*_*f*_ value indicates a more tenuous aggregate. Observation of the fractal aggregation process can further be used to control whey protein-based aggregation so that foods with uniform and appropriate texture can be produced [8].

There are several techniques that can be used to analyze fractal structure in aggregates. These experimental methods were widely used to analyze and evaluate texture characteristics of whey protein-based foods which are carried out by forming a model of whey protein gel system that can be used as medium. The measurement of this physical quantity is related to mass distribution in space and can be done through various techniques, such as scattering, settling, microscopy (image analysis), and rheology [9, 10]. This paper will only limit the discussion on two methods: microscopy and rheology. These methods, however, have several limitations which might affect the fractal dimension values and type of aggregations obtained. The review is therefore divided into three sections; the first section discussed the structure of whey protein and its functionality; the second section is about fractal theories in relation to food structure, methods for fractal analysis, and their limitations; the third section is a compilation on fractal dimension values and the types of aggregation of whey protein aggregates gathered from several researches. This was done to finally understand how the texture of whey protein-based food is formed despite the limitations available on each method.

## 2. Structure and Functionality of Whey Protein

### 2.1. Composition and Structure

**Figure 1:**
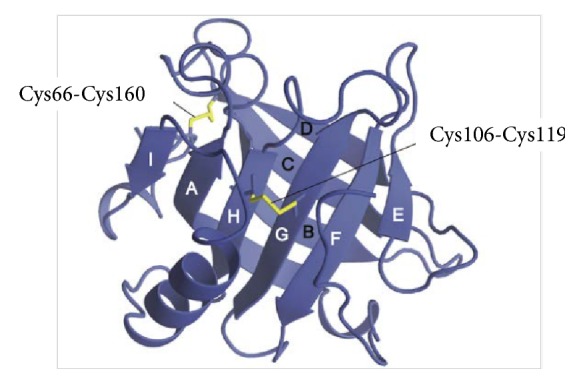
*Structure of β-Lg*. Yellow lines represent disulfide bonds (adapted from Ikeguchi [89]).

Proteins provide various functions in food and also maintain the stability of food structure. Whey proteins can interact with each other and with other types of protein to make networks associated with gels. Whey protein comprises a globular structure as shown in Figure 1. Globular proteins are usually curled up so that the hydrophobic R regions are centered in the molecule to avoid the polar environment around them, while the hydrophilic R group is located on the surface of the molecule. This makes globular proteins (i.e., whey protein) soluble in water. *β*-Lg as a major component in whey protein determines the properties of the overall whey protein. Each *β*-Lg molecule has 5 cysteine (amino acid) residues: Cys-66, Cys-106, Cys-119, Cys-121, and Cys-160. Cys-66/Cys-160 and Cys-106/Cys-119 are connected by disulfide bonds (-SS) to form oxidized form of cysteine, that is, cystine, while Cys-121 is available as a free thiol (-SH) group. One molecule of *β*-Lg (whey protein), therefore, consists of one free thiol group and two disulfide bonds [11, 12]. The free thiol group is entrapped in the three-dimensional structure of* native* whey protein and exposed during denaturation. These structures are important in terms of protein aggregation [13]. Heat treatment, high pressure, mechanical stress, and oil-in-water exposure to air during food processing can cause changes in the tertiary (globular) structure of the whey protein so that the thiol and cystine groups become exposed to the solvent and become chemically reactive [12].

### 2.2. Whey Protein as Gelling Agent

Whey protein can enhance texture formation in food due to one of its functionalities to form gel. This is an important functional attribute to many food applications, such as meat and milk processing, and bakery, and also to improving appearance of food products such as yogurt [1, 4, 14].

**Figure 2:**
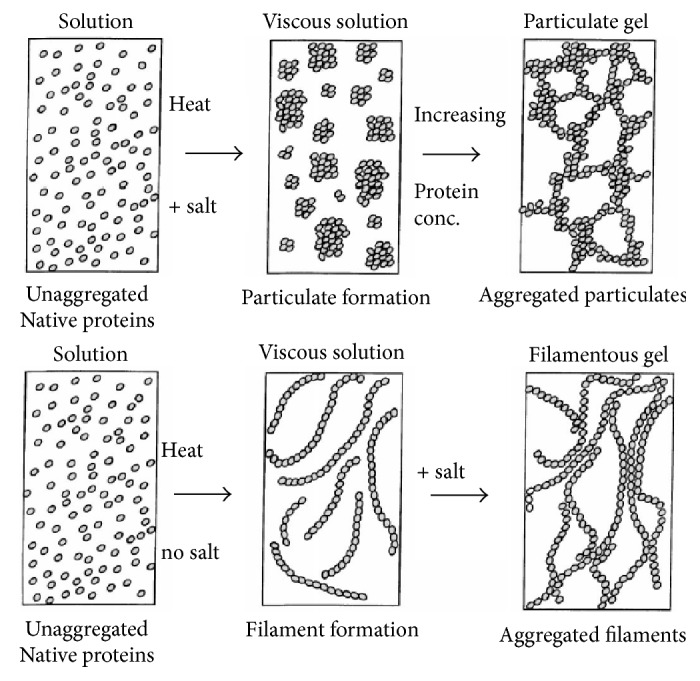
*Illustration of gel formation* (adapted from Bryant and Julian McClements [15]).

Rheological properties of protein gel vary depending on the type of protein, pH, ionic strength, and rate of heating. Gel of globular protein is classified into two distinct types of morphology: particulate and fine-stranded gels. Extensive reviews on these morphologies were done by Bryant and Julian McClements [15] and Nicolai and Durand [6]. In general, particulate gels are turbid gels that can be formed by modifying the pH close to the isoelectric point or at high ionic strength (low electrostatic repulsion force), while fine-stranded gels are transparent filamentous gels that can be formed at pH far from the isoelectric point in the absence of salt or low ionic strength (high electrostatic repulsion force).  Figure 2 showed the microstructure on each type of gel.

Verheul and Roefs [5] stated that there are three phenomena involved (often subsequently) in aggregation process of globular protein, that is, conformational changes, chemical reaction, and physical interaction. Conformational changes are related to denaturation process and consist of two steps. The first step is* unfolding *of globular protein which is then followed by the second step: aggregation [1]. Proteins in the native form initially undergo a process of structural conformation changes (denaturation) due to modification of the external environment [16]. The* unfolding *of globular whey protein causes the exposure of hydrophobic, free thiol, and disulfide groups which are initially present in the interior of globular protein, thus allowing inter- and intramolecular interactions among whey proteins [15]. This exposure leads to chemical reactions and aggregation through covalent and noncovalent bonds [5, 7, 8, 11, 15, 17–19]. The aggregation process itself is divided into two stages: primary aggregation that leads to the formation of spherical particles or flexible strands (filament) and secondary aggregation that occurs as protein and salt concentration increase. This secondary aggregation finally leads to formation of gel, precipitate, or fractal clusters [6]. A further stage of aggregate formation is physical interaction or commonly noticed as cluster-cluster aggregation. Particles in this stage diffuse and stick together randomly in certain media with certain probability and form larger clusters (gel network) [20]. The process of gel formation by whey protein itself can be done in two ways, namely, heat and cold gelation.

*Heat Gelation.* Heat gelation is done by heating whey protein solution sample at gelation temperature until denaturation and aggregation occur. The gel is prepared by heating the native whey protein solution (*C*_*g*_ = 2–200 g/L, temperature > 60°C) for 10 minutes to 24 hours at various pH (2.5–9.0) with certain salt concentration (~20 mM–1.0 M NaCl) [15, 18, 21–28] followed by rapid cooling at room temperature [1]. McClements and Keogh [29] observed that the process of gel formation in heat gelation is influenced by temperature and heating time. The gelation temperature of aggregated whey protein was 48°C while for native whey protein it was 77°C. This is because the aggregated whey protein has more exposed hydrophobic groups to the whey protein solution. Hydrophobic interactions that occur can therefore overcome the electrostatic repulsion forces at lower temperature so the gelation temperature is also lowered. On the other hand, the gel will not form on native whey protein solution until the temperature reaches a point where unfolding occurs. This effect of heating is also influenced by pH, ionic strength, and the concentration of salt, protein, sugar, and fat [1, 14]. Aggregation and gelation in heat gelation also occur simultaneously [13].

The formation of covalent bonds in the form of additional disulfide bonds from the oxidation of thiol groups or thiol-disulfide exchange reaction occurs in both gelation types. The formation of additional disulfide bonds stabilizes some weak clusters and decreases the potential for restructuring during gel formation [30]. Hoffmann and van Mil [31] examined the role of free thiol groups and disulfide bonds in the heat gelation of *β*-Lg; the result showed that *β*-Lg is dispersed at neutral pH and heating at 65°C enhanced the formation of aggregate mainly due to presence of intermolecular disulfide crosslink.

*Cold Gelation*. Cold gelation consists of two stages: the preparation of heat-denatured whey protein solution and gelation at low (or ambient) temperature. In contrast to Verheul and Roefs [5], research by Alting et al. [32] indicated that the initial stage in cold gelation process was physical interaction. This stage was then followed by an increase in hardness and stiffness of the gel due to a covalent reaction between structural elements of the gel. Aggregation and gelation process occur separately throughout this process. This causes the properties of the gel in cold gelation to be readily adjusted in the early stages of aggregation before the gelation [13, 15].

The aim of preparing a heat-denatured whey protein solution is to produce a solution containing filamentous type of protein aggregate that does not gel [15]. This is done by heating the native whey protein solution at low concentration (*C*_*g*_ = 6–10% (w/v)) [29, 32–38], at temperatures between 70 and 90°C for 5–60 minutes [29, 30, 35], at pH distant from the isoelectric point (~2 units above the pI of protein), and at low ionic strength (<50 mM NaCl or <10 mM CaCl_2_ at pH 7 [29, 35, 36, 39–43]) to form soluble aggregates. The process of gel formation is influenced by a combination of protein concentration, pH, ionic strength, temperature, and heating time. The gel is formed on a critical gel concentration (*C*_*g*_), where at a concentration higher than *C*_*g*_ the gel network will not flow if tilted but will flow at a lower concentration. *C*_*g*_ will decrease with increasing salt concentration and decrease in pH, while aggregate size will increase [30]. The next step is to induce gelation at room temperature by adding salt (salt-induced) NaCl [8, 38] or CaCl_2_ [8, 35, 38, 42]. Higher NaCl concentrations are needed to induce gel formation from cold gelation process than CaCl_2_ concentrations [44, 45]; this is because calcium has a specific ion bridge effect that contributes to gel formation. The protein concentration which is typically used for salt-induced cold gelation is 100 g/L, where the rate of aggregation after salt addition increases sharply with increasing salt and protein concentration. The rate of aggregation also increases with increasing temperature due to exposure of hydrophobic groups that lead to hydrophobic interactions [29, 30, 44]. Kuhn et al. [46] suggested that the use of different types of salt encourages the formation of different gel structures. Gelation with CaCl_2_ produced irregular microstructure (particulate) resulting in stronger, more elastic, and turbid gels, whereas gelation with NaCl produced gel with a more-ordered microstructure but more brittle.

Gelation also can be performed by adjusting pH of the heat-denatured solution to the isoelectric point of protein. This is commonly called acid-induced cold gelation, one type of reagents which were used frequently is glucono-*δ*-lactone (GDL) [32–34, 47–50]. The addition of acid leads to a decrease in electrostatic forces on proteins so that aggregates are formed. Stronger gel is generally produced by acid gelation rather than by calcium gelation [48, 49, 51], where maximum strength is achieved at pH ~ 5 which is the isoelectric point of *β*-Lg [13, 47, 49]. The morphological properties of the initial gel network of acid-induced cold gelation are formed as a result of noncovalent bonding [52]. The formation of additional disulfide bonds stabilizes some of the weak clusters and decreases the potential restructuring during gel formation. The formation of disulfide bonds in acid-induced cold gelation had also been studied by Alting et al. [32, 33, 52]; the formation of disulfide bonds in this type of cold gelation was surprising since oxidation of thiol groups or thiol-disulfide exchange reaction generally occurs under alkaline conditions [15]. The results showed that the formation of additional disulfide bonds may occur, depending on the pH used during the process, in which the disulfide bonds cannot form at pH 2.5–3.5. The rate of acid gelation also controls the level of structural rearrangements during gel formation and finally affects the properties of the gel. The acid gel texture after gel formation tends to be unstable, and hardening gradually occurs due to thiol-disulfide exchange reaction during storage at a pH higher than 3.9 [53].

There were different insights regarding the role of thiol-disulfide exchange reaction in acid-induced cold gelation. Famelart et al. [54] also stated that thiol-disulfide exchange reaction during acid-induced cold gelation also prevented the formation of large covalent structures during gelation of acid under pH 5; the result also suggested that the role of thiol-disulfide exchange reaction during acid gelation at various pH was almost insignificant. This was demonstrated by the insignificant difference of elastic modulus of milk gel formed by heating reconstituted milk with and without addition of thiol-blocking agent (N-ethyl-maleimide or NEM). This was in contrast with Vasbinder et al. [55], Lucey et al. [56], and Lucey et al. [57] that showed significant differences in elastic modulus and gel hardness made with or without the addition of thiol-blocking agent, in which the gel with the addition of thiol-blocking agent has a slightly modulated (~20%) elastic modulus and gel hardness (~30%) after gelation. This clearly showed the presence of additional disulfide bonds during and after gelation. Relatively different results were shown by Cavallieri et al. [47], in which the disulfide bond is associated with the internal stabilization of whey protein aggregates formed only during the initial heating in acid-induced cold gelation.

## 3. Fractal Concept and Quantification Methods

**Figure 3:**
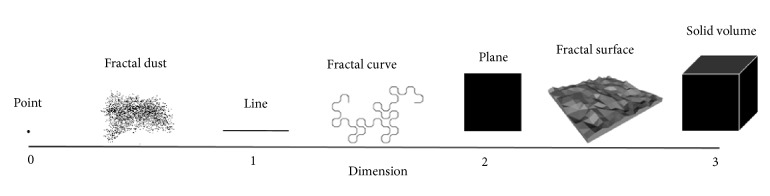
Illustration for conventional (Euclidean) and fractal dimensions.

Many foods, like many other natural materials, are inherently irregular in conformation. Food has a complex geometry in which a large category of structural irregularities exists, including pores (bread, snack, and cereals), protuberances (cauliflower), and replicating structures (broccoli). Such attributes may exist over wide levels of magnification. Fractal concept was first introduced by Mandelbrot [58] to describe dimensions between regular or conventional dimensions 1, 2, and 3. Fractal dimension indicates the degree to which an image or object deviates from regularity (Figure 3). A feature of mathematically constructed fractal objects is self-similarity: the attribute of having the same appearance at all magnifications or length scales. Natural objects like food therefore can be characterized quantitatively in terms of their fractal dimension, which may serve as an index of irregularities [59]. The fractal model may describe particle aggregation in a mixture of both particles, from the point when acidification renders the particles reactive [21] to the point when clusters have grown enough to come in close contact, interpenetrate, and percolate. The fractal dimension (*D*_*f*_) is used to describe the occupancy of the structure in the volume of the gel, while the scaling behavior can give insight into the mechanism of assembly of the particles. Fractal analysis explains the quantitative analytical method that characterized disorder shape of particles network of colloidal system [58]. The fractal concept has led to considerable advances of our understanding of colloid aggregation process including the structure and size distribution of the aggregates as well as the kinetics [60].

### 3.1. Fractal Dimension in relation to Types of Aggregation

Gel is an elastic network formed by collection of crosslinked macromolecular chains that interact with each other. Physical interaction processes (cluster-cluster aggregation) which occur in gel formation are greatly affected by one of the main characteristics of particle, namely, Brownian motion. This motion occurs since the small size of particles causes unequal collisions. As a result, the particles change direction and produce a zigzag random motion. The Brownian motion allows particles to overcome the effect of gravity so that the particles do not settle out or separate from the dispersing medium.

**Figure 4:**
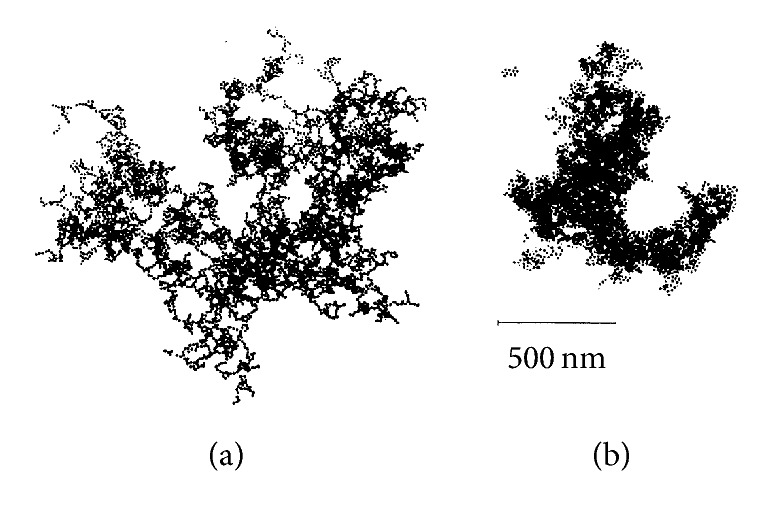
*DLCA (a)*;* dan RLCA (b)* (adapted from Weitz et al. [61]).

The growth model or ideal aggregate formation of particles network based on Brownian motion is divided into two limiting regimes: diffusion-limited (DLCA) and reaction-limited cluster-cluster aggregation (RLCA) (Figure 4) [8]. The fractal concept can be used to explain the fractal aggregation regimes of DLCA and RLCA by quantifying the structure of aggregated particles by using a parameter, namely, fractal dimension (*D*_*f*_). Aggregation on DLCA regime is very rapid and the collisions among particles are limited only by Brownian motion. The aggregate formed on this regime is indicated by *D*_*f*_ value of 1.7–1.8. Meanwhile, aggregation in RLCA regime occurs more slowly due to the electrostatic repulsions between approaching particles. Aggregates formed under these conditions are marked with slightly higher *D*_*f*_ (2.0–2.2). Weitz et al. [61] also stated that aggregate formation with *D*_*f*_ value above 2.0 was irreversible so that it could be best described as in RLCA regime. The *D*_*f*_ values represented in both regimes were also proven to have important effects on the mechanical properties of aggregated network [8, 10, 61–63].

### 3.2. Scaling Theory

**Figure 5:**
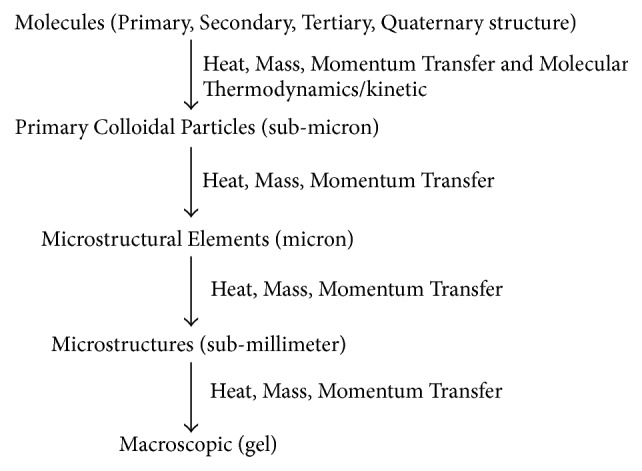
*Mechanism of protein gel formation* (adapted from Marangoni et al. [38]).

The macroscopic nature of gel is somehow affected by hierarchical level of various factors, which can be attributed to the properties of each individual protein molecule. The importance of this structural hierarchy cannot be ignored (Figure 5). They physicochemical properties of individual protein molecules as well as environmental conditions (pH, temperature, ionic strength, etc.) will affect the type of primary particles formed, interactions between particles, and ultimately the final three-dimensional network structure. The observed macroscopic properties of the system are influenced by various levels of structure in the hierarchy; the most influencinglevel is definitely the one closest to the macroscopic level, that is, the microstructure level. Prediction and manipulation of the macroscopic nature of the gel therefore require an understanding of the effect of properties present at the microstructure level on the macroscopic properties of gel. Not involving the microstructure level will undoubtedly lead to failure in predicting and manipulating macroscopic properties of gels [38].

There are several developing methods for determining fractal dimension values of complex material such as whey protein gels, namely, scattering, settling, image analysis, and rheology. This review will only limit its discussion to two methods: image analysis and rheology. This is also based on the fact that the gel aggregate is a quantifiable fractal structure from rheological and optical measurements [38].

### 3.3. Fractal Structure Measurement

#### 3.3.1. Microscopy (Image Analysis)

Interpretation of rheological and mechanical properties depends on the structural information derived primarily from microscopic methods, image analysis, and the use of computer simulations to test the model of food structure, in which rheological techniques cannot provide direct information about the underlying food structure at molecular level. This is because most of the important elements affecting physical and rheological properties as well as texture and sensory properties are available in sizes below 100 *μ*m. The development of the first (optical) microscope opens a new way to visualize and describe material structure at the molecular level. Microscopic techniques have the same significance as rheology techniques and are available for analyzing food structure at different hierarchical levels. The use of microscopic methods to study food can reveal additional structural information and new insights and applications in food science and technology [9, 64, 65].

Image analysis works well for large particles with high contrast and low dimensionality. High contrast and large particles are the attributes needed to produce a good and clear image, so that the important structural information can be extracted from the image. The whey protein aggregate of 2D images is obtained from several types of microscope: transition electron microscope (TEM) [34, 38], scanning electron microscope (SEM) [46], and confocal scanning laser microscope (CSLM) [21, 32, 34, 60, 66–69]. Image taken from CSLM should be in 2D format, meaning that the Z-stack should not be applied. The images will further be processed by using image processing software (*ImageJ*,* Fiji*, etc.). Through the software, images with certain resolution are converted to grayscale format and then to binary color (black and white). Determination of threshold value (*T*_*L*_) is therefore important as a first step before the image is converted to binary color. Thresholding is performed to determine whether a pixel of a given intensity (0–255; 0 = black, 255 = white) in the image is considered as an object or background. For the image taken from CSLM, the concentration of protein label, that is, Rhodamine B, should be added by considering the total protein concentration in the sample; otherwise the unbound Rhodamine B will appear as an artefact.

Several methods to determine *T*_*L*_ of aggregate images were proposed by Kuhn et al. (2010), Pugnaloni et al. (2005), and Thill et al. (1998) [46, 70, 71]. After conversion to binary image, the *D*_*f*_ value from the image is then obtained by using box counting method (BCM), in which various sizes of grids are placed on the image and the number of boxes containing the pixels of object (*N*) is calculated on each grid size (*L*). The log-log graph between *N* as ordinate and *L* as abscissa is then plotted and the slope of the graph is a fractal dimension value in two-dimensional space (*D*_BCM_). The *D*_*f*_ value in three-dimensional space which represents the gel aggregate structure is determined as follows: *D*_*f*_ = *D*_BCM_ + 1 [9, 72].

*Limitations on Image Analysis Technique*. Andoyo et al. [34] observed the microstructure of mixture of whey protein aggregates and native micellar casein by using both CSLM and TEM. The results showed that the *D*_*f*_ value from CSLM image is slightly lower (2.64) than the TEM image (2.69), although both results were still in the same aggregation regime (RLCA). Hagiwara et al. [60] observed images of *β*-Lg and BSA gels made by heat gelation method in the absence of salt at pH 7.0 by using CSLM. The resulting aggregate image is not clear; the researchers proposed that it occurred because the size of the aggregate was too small to be observed by microscope. This also happened in the research done by Andoyo et al. [34] which used TEM to observe acid-induced cold-set gel in whey protein aggregate system—the *D*_*f*_ value cannot be extracted from the image since the floc was so small that the image had a low contrast and *T*_*L*_ cannot be determined. The advantages and disadvantages of image analysis from different types of microscope for dairy gels had been explicitly described by Ercili-Cura [73]. In general, CSLM, which was widely used to analyze the whey protein aggregate images, is capable of projecting three-dimensional structure of gel unto two-dimensional plane (image). The initial sample handling for CSLM is also easier compared to the other microscope, so it does not trigger the structural changes of the gel. CSLM can also provide a more homogeneous luminous flux on each observed image so that the intensity of the background and object colors can be clearly distinguished. On the other hand, SEM and TEM produced a higher resolution image compared to CSLM, but initial sample preparation is so rough and complicated that it might trigger structural and morphological changes of gel aggregates.

In addition, determination of *T*_*L*_ also affects the *D*_*f*_ value obtained from an image. Ako et al. [66] applied different methods of image analysis. Various gray level intensities as *T*_*L*_ in *β*-Lg gel image, higher *T*_*L*_, resulted in lower *D*_*f*_ value and vice versa. In higher *T*_*L*_, more pixels of the image are considered as part of the background. The effect of changing thresholding led to a change in the contrast and thus change of the *D*_*f*_ value. The *D*_*f*_ value was also determined by the rhodamine concentration; it should be sufficient to give a proper signal. However, too much rhodamine added could change the structure. Furthermore, it was not satisfied with the self-similar concept. The exact *T*_*L*_ value therefore greatly determines the accuracy of *D*_*f*_ value and aggregation regime obtained. In addition to taking a long time and inconsistency, different results are likely to be obtained on different time or by different operators. Manual thresholding errors cause more problems during analysis compared to the other causes [74]. This was also supported by Andoyo et al. [34] who used three different manual thresholding methods [46, 70, 71] on mixture of whey protein aggregates and native micellar casein image, in which the result showed that different proposed manual thresholding methods resulted in relatively distinct values of *T*_*L*_ and *D*_*f*_. Russ [74] suggested that the use of automated thresholding methods is therefore more advisable. These automated thresholding methods are readily available in the image processing software based on several algorithms proposed by many image processing researchers [75, 76]. These algorithms are based on the information or knowledge about the subject and images and how the images are acquired. However, the different types of automated thresholding algorithms that were previously proposed by many researchers also provide different binary images. Further objective and quantitative evaluation is needed to determine the exact algorithm for each type of image. Sezgin and Sankur [77] performed a quantitative evaluation based on the average value of 5 criteria against 40 algorithms for automated thresholding. These algorithms were applied to document images and also to the nondestructive testing images (NDT), in which there were 6 images produced from light microscope with bimodal histogram distribution [78]. The result showed that the minimum error method algorithm proposed by Kittler and Illingworth [75] produced the most uniform and accurate binary image among other methods. This result was also supported by previous evaluation done by Glasbey [79]. To the authors' knowledge, this automated thresholding algorithm has been applied to images of whey protein gel but it should be optimized; furthermore the use of this algorithm for whey protein aggregate image with bimodal and multimodal [80] histogram distributions is quite promising for certain type of gels. For images with unimodal histogram distribution, statistical analysis of geometric parameters can be done prior to image processing as stated in Chu et al. [81] or Silva et al. [82]. Thresholding of the images can be optimized by using automated methods for certain types of gel. For a certain type of gel, the calculation of *D*_*f*_ is sensitive to the threshold value. This is the reason why an optimized method was used. Variations were also observed when manual thresholding of images with a wide range of threshold values was applied. Nevertheless, changing the threshold level or using different thresholding method does not really change the message.

#### 3.3.2. Rheology

Determination of *D*_*f*_ value using rheological properties of gel requires a model that covers the relationship between the structure of the gel with its rheological properties; the most appropriate model for gel aggregate structures with self-similar pattern is based on scaling theory. The early development of scaling theory to explain the elasticity of gel was proposed by Buscall et al. [83] who proposed that aggregates network is fractal on a scale greater than their primary particle size and formulated the power-law relationship of elastic modulus (*E*) to solid volume fractions. The value of *E* is equal to particle concentration of *φ*^*A*^ and the value of strain at limit of linearity (*γ*_0_) is equal to particle concentration of *φ*^*B*^ which are then linearized to

(1) log⁡E=Alog⁡φlog⁡γ0=Blog⁡φ,

where *A* and *B* are the slopes of log-log plot between rheological parameters and particle concentration. Exponents *A* and *B* will vary with different gel systems [32]. The calculation of fractal dimensions by means of rheological method is generally applicable to various rheological parameters of a material, including strain at limit of linearity (*γ*_0_) [20, 32, 34, 38, 60, 68]; storage modulus (*G*′) [8, 21, 32, 34, 84]; elasticity (*E*) [38, 60, 68]; and shear stress (*σ*) [8]. These various rheological properties of gel aggregate can be determined by using rheometer or texture analyzer. The power-law theory was further verified by many researchers, so three models of scaling theory were presented: Bremer [85], Shih et al. [63], Wu and Morbidelli [10]—these three scaling models are basically used to find the relationship between rheological properties of a gel and its network structure. Bremer [85] classified the particles of gel network into two types: straight strands and curved strands (Table 1). Shih et al. [63] classified the gel network into strong-link and weak-link regime (strong-link: the extent of viscoelastic linear region decreased as increasing protein concentration and vice versa (Table 1)), based on the strength of inter- and intrafloc interactions, while Wu and Morbidelli [10] added another regime, that is, transition regime to describe the intermediate situation where both inter- and intrafloc interactions give similar effect to the gel elasticity. Furthermore, Narine and Marangoni [86] proposed the mechanical models for colloidal aggregates that relate the fractal dimension of a colloidal aggregate to mechanical properties, as follows:

(2) $$\begin{array}{c} G^{\prime }\sim \frac{mA}{6c\pi \sigma \xi d_{0}^{3}}\Phi^{1/\left(d-D\right)}, \end{array}$$

where *G*′ is elastic modulus, *m* is spring constant, *c* is proportionality constant, *A* is Hamaker's constant, *ξ* is diameter of the microstructure, *σ* is the diameter of a microstructural element assumed to be spherical, Φ is the volume fraction, *d* is the Euclidean dimension of the network, usually 3, and *D* is the fractal dimension of the network. They found that the model successfully identifies key parameters that are important in determining the fractal dimension value. The model relates the values of Hamaker's constants and size of microstructural elements with the composition of the particles network. Furthermore, this model follows the weak-link theory in which the rheological parameter of the network is dependent on the nature of the link between microstructure as opposed to the strength of the microstructures themselves and only valid for the relatively high percentages of solid content (60–100%).

Mellema et al. [87] proposed five types of gel structures, namely, random, curved, hinged, straight, and rigid, which could be derived using the framework of Kantor and Webman. This model is generalized by introducing a scaling parameter, *ξ* as percolation length, *δ* as a measure of the number of deformable links in a strand, and a parameter *ε* as a measure of the bendability of the link. The model is shown as follows:

(3) G′∝ξ−1+2ε+δγ0∝ξ2ε+δ−1σ∝ξ−2.

This model can at least cover a wide range of gel types by using three rheological parameters; the storage modulus, the yield stress, and the maximum linear strain as a function of the volume fraction. Table 1 summarizes these three scaling models.

*Limitations on Rheological Technique. *Ikeda et al. [20] used the scaling model of Shih et al. [63] that showed that the value of *D*_*f*_ for heat-induced WPI gel produced at 25 mM, 100 mM, and 500 mM NaCl decreased, except at 100 mM NaCl. This suggested that WPI gel at given NaCl concentration (100 mM NaCl) cannot be predicted appropriately by the fractal model. The study also showed that limit of linearity cannot be used to determine the value of *D*_*f*_ since it produced unreasonable value (*D*_*f*_ between 0.2 and 0.7). The limit of linearity parameter in this study was only used to determine the regime classification of the resulting gel in this study. Research done by Alting et al. [32] on acid-induced cold-set WPI gel using scaling model of Shih et al. [63] and Bremer [85] also yielded ambiguous *D*_*f*_ values. This ambiguity also occurred in heat-induced *β*-Lg gel at various NaCl concentrations in the research done by de Kruif et al. [21]. The use of different scaling models can also result in different *D*_*f*_ values. This significantly affects the interpretation of the resulting aggregation regime. Although rheological technique is relatively easy to apply and can be performed at higher range of particle concentration, this method has limitation in terms of appropriate scaling model and rheological parameters. The use of scaling models and rheological parameters under different gel conditions determines the accuracy and consistency of the resulting *D*_*f*_ values.

Another limitation includes the following: rheological data from various instruments such as rheometer or texture analyzer cannot directly generate strain values at limit of linearity; this leads to the need for further processing of data manually to determine the value. Hagiwara et al. [60, 67, 68] defined limit of linearity as the value of strain in which there was a deviation of 5% (see (2)) between the ordinate value (*σ*) of stress-strain curve with *γ (strain)* ×  *E* (elasticity). The elasticity value itself was the slope value of linear part of stress-strain curve at *γ* < 0.01. Andoyo et al. [34] also defined limit of linearity the same way but formulated the calculation for deviation (see (5)) in a relatively different way, in which there was a deviation of 5% between slope at the end of linear part of stress-strain curve (*R*^2^ ~ 0.99) (*slope*_*o*_) with slope at certain point (*slope*_*n*_) after the end of linear point of stress-strain curve.

(4) DeviationH=σ−γ×Eγ×E×100%

(5) DeviationA=slopeo−slopenslopeo×100%

(6) Limit  of  linearity=strain  γ  value  at  5%  deviation.

Each method used to determine the value of deviation and limit of linearity is relatively subjective; therefore each produces different value of limit of linearity. The use of 5% deviation value is also determined subjectively by the researchers so that different percentages of deviation will also result in different limit of linearity values. This was supported by Andoyo et al. [34], where the use of 5% and 10% deviations in determining the strain at limit of linearity yielded relatively different values. Hagiwara et al. [67] also indicated that the slope value of log⁡*γ*_0_ and log⁡*φ* could not be used to find *D*_*f*_ values of the observed gel system; this might due to the nongenerality of method used to determine the value of limit of linearity or the 5% deviation value used was less accurate to determine the value. In some cases, the fractal assembly of whey protein gels only occurred in the suspensions at the early stages of the acid gelation/aggregation, until the flocs started to come in contact, interpenetrate, and eventually percolate into a gel [34, 88]. Therefore, the range of length scales where acid gelation self-similarity investigated was ranging from ~0.1 to ~10 *μ*m. Fractality may also exist in whey protein-containing samples below the 0.1 *μ*m length scale and it is possible that the flocculation mechanism somewhat differed among different samples tested [88]. In another case, acid cold gelation probably starts off as a fractal process but is rapidly taken over by another mechanism at larger length scales (>100 nm) [32].

## 4. *D*_*F*_ Value of Whey Protein Aggregates

### 4.1. *D*_*f*_ from Microscopy

This section will explain all the *D*_*f*_ value compilations from various researchers by using microscopy method. de Kruif et al. [21] examined the gel from the same solution but with addition of 0.1 M and 0.5 M NaCl heated at 68.5°C. Hagiwara et al. [60, 67, 68] analyzed gels made from BSA solution with the addition of 0.1 M NaCl (pH 5.1), 30 mM CaCl_2_ (pH 7.0), and 5 mM CaCl_2_ (pH 7.0) which were heated at 50°C for 60 minutes followed by 95°C for 10 minutes. In addition, observations were also made on the gel made from the *β*-Lg solution with the addition of 1.0 M NaCl at pH 7.0 and 30 mM CaCl_2_ at pH 7.0 which were heated at 40°C for 60 minutes and then 95°C for 10 minutes. All gels formed from those various researches were then observed by microscopy method. The *D*_*f*_ values generated from this method for heat gelation of whey protein and its components are in the range of 2.2–2.81. The addition of salt and adjustment of pH of the protein solution prior to heating process to regulate the ionic strength of the solution also affected the value of *D*_*f*_, where *D*_*f*_ decreased with increasing ionic strength. Higher *D*_*f*_ values may occur due to restructuring and micro-phase separation process occurred in the protein gel during aging (storage) [6, 32, 68]. The image from CSLM also showed that the gel structure tended to be more homogeneous at NaCl concentrations below 0.2 M and more heterogeneous at higher ionic strengths. Gel formed from whey protein solution with salt concentrations higher than 0.2 M had a more heat-sensitive structure, whereas at lower ionic strength the heating temperature only affected the kinetics of gelation [21, 66]. The *D*_*f*_ value derived from the microscopy method for whey protein gel made from the heat gelation method lay between 2.2 and 2.7 in various types of conditions so that it could be ideal for reaction controlled gel formation. However, such high range of *D*_*f*_ values could lead to a different structure formation mechanism as it is not fully complying with well-defined RLCA model.

Marangoni et al. [38] studied salt-induced cold-set WPI gels with 10% (w/v) protein concentration induced by CaCl_2_ (10, 30, and 120 mM) and 9, 10, 11, and 12% (w/v) protein concentration induced by 300 mM NaCl. The heating was carried out at temperature of 80°C for 30 minutes before gelation at room temperature. Kuhn et al. [46] also studied gel with the same gelation process in 10% (w/w) WPI concentration which was heated at 90°C for 30 minutes and then diluted to 5, 6, 7, 8, and 9% (w/w) WPI concentrations. These solutions were then induced by 150 mM NaCl or 150 mM CaCl_2_ to form gel. The *D*_*f*_ values for these salt-induced cold-set whey protein gels were in the range of 2.45 and 2.81 for NaCl-induced and 2.63 and 2.82 for CaCl_2_-induced gels. The difference in values resulting from both studies can be due to differences in rate of aggregation, time required to achieve equilibrium conditions, or heating temperatures [46]. Marangoni et al. [38] also stated that microscopy method yielded *D*_*f*_ values similar to rheological technique at NaCl and CaCl_2_ concentrations above 30 mM. The *D*_*f*_ values obtained for whey protein gel prepared by the salt-induced cold gelation method from various studies under various conditions were between 2.25 and 2.82.

The images from SEM showed that the cold-set gel induced by CaCl_2_ had a thinner, more compact microstructure, with a less porous network. As WPI concentration increased, the number of pores decreased and the gel network formed had a denser structure; this could be associated with an increase in water-holding capacity (WHC). Cold-set WPI gels induced by NaCl represented a more porous network but had a higher WHC than gels induced by CaCl_2_; this might be because the WHC depended not only on the porosity of the gel, but also was influenced by the polymer characteristics (its availability in water binding) which was highly dependent on the types of salt added. NaCl-induced gels were transparent (fine-stranded) while CaCl_2_-induced gels were turbid (particulate). Fine-stranded gels tended to have higher WHC since they contained smaller and more homogeneous pore sizes that can bind water more strongly [15, 46, 47]. The increased salt concentration also caused the gel to become more turbid; this suggested an increase in the size of the particle diameter as the salt concentration increased at constant *D*_*f*_ value. Increased protein concentrations led to more transparent gels; this indicated a decrease in particle size as the concentration of protein increased at constant *D*_*f*_ value [38, 50].

Alting et al. [32] compared gels formed from 9% (w/w) WPI heated at 68.5°C and induced by GDL with and without addition of thiol-blocking agent NEM. Andoyo et al. [34] observed *D*_*f*_ values in WPA (whey protein aggregates) made from 90 g protein/kg WPI solution heated at 68.5°C, pH 7.5, for 2 hours and then standardized to 70 g protein/kg. *D*_*f*_ observations were also performed on mixture of WPA and NMC (native micellar casein) with a ratio of 20 : 80. Both types of solution were then set to achieve certain concentrations (14–62 g protein/kg for WPA and 15–90 g protein/kg for WPA and NMC mixtures). The gel formation was then induced by using GDL. The *D*_*f*_ values generated from microscopy method for acid-induced cold-set gels were in the range of 2.15–2.69. The results were consistent with other studies that suggested that *D*_*f*_ values for gels induced by using GDL or microorganisms were in the range of 2.3–2.4 [85]. Eissa and Khan [90] compared *D*_*f*_ values obtained from gels made from transglutaminase-modified WPI and WPI without modification. WPI solution at various concentrations and pH 7.0 was initially heated at 80°C for 1 hour and transglutaminase enzyme was added at 50°C while cooling, stirred for 20 minutes, and incubated at the same temperature for 10 hours. The gel formation was then induced by using GDL at room temperature until pH increased to 4.0. The resulting gel based on the CSLM image showed that both types of gel had fine-stranded morphology with *D*_*f*_ values of 1.96–1.98.

The microscopy method has even been used not only for whey protein-based gel models, but also for comparing the structure of protein-based food products. Torres et al. [69] substituted the use of fats in yogurt by using several types of microparticulate whey protein (MWP) with different nutrient compositions (including different amount of native and denatured whey protein). The results showed that the *D*_*f*_ values were in the range of 1.4–2.6, in which lower amount of native whey protein would form less interconnected network with low self-similarity. MWP with higher amount of native whey protein produced yogurt with characteristics similar to that of high-fat yogurt. This suggested that the denatured whey protein might act as a structure breaker because of its inability to form a cohesive network. The appropriate MWP types therefore could substitute the use of fats in yogurt production. Detailed data presented above was collected in Table 2.

### 4.2. *D*_*f*_ from Rheology

This section will discuss all the *D*_*f*_ value compilations from various researchers by using rheology method. Stading et al. [84] conducted a study of *β*-Lg gels made by heat gelation method. The *β*-Lg solution at pH 5.3 or 7.5 was heated at a rate of 0.1°C/minute or 5°C/minute from 30 to 90°C and was held constant for an hour. Microstructures formed on rapid-heated gels were more open and inhomogeneous and had a higher fracture stress; meanwhile the slow-heated gels had more homogeneous and compact microstructure. This was also supported by *D*_*f*_ value generated from the same study by using scaling model of Bremer [85] with rheological parameter *G*′, where gels formed at pH 5.3 had a particulate morphology with *D*_*f*_ value of 2.46 for 0.1°C/minute heating rate and 2.46 for 5°C/minute. Gels at pH 7.5 were fine-stranded with *D*_*f*_ value of 2.94 for 0.1°C/minute and 2.91 for 5°C/minute. Hagiwara et al. [60, 67, 68] also examined the *β*-Lg and BSA gels made by heat gelation by using scaling model of Shih et al. [63] and elasticity as rheological parameter to determine *D*_*f*_. BSA and *β*-Lg solutions with various protein concentrations were initially added with 50 mM buffer, pH 7.0, or 50 mM acetate buffer, pH 5.1, and then varied with or without salt addition (0.1 M NaCl or 5 and 30 mM CaCl_2_). The solutions were then heated at 40–50°C for 60 minutes, followed by a temperature of 95°C for 10 minutes, and cooled to 25°C and stored for 24 hours. The addition of NaCl and CaCl_2_ salts to the protein solution prior to heating caused the gel to be classified in the weak-link regime with *D*_*f*_ values between 2.61 and 2.82, whereas the gel formed without the addition of salt at pH 7.0 fell into the classification of strong-link regime with *D*_*f*_ values between 2.00 and 2.20. Hagiwara et al. [67] also more specifically suggested that the *D*_*f*_ values resulting from BSA gel aggregates were greater than *D*_*f*_ values of aggregates in the aqueous solution (results gathered from scattering method) [91]. This might be due to the interpenetration effect between aggregates at high protein concentration to form a compact gel (higher *D*_*f*_ value). de Kruif et al. [21] examined the gel from WPI at various concentrations added with 0.1 and 0.5 M NaCl prior to heating (heat gelation). Heating was done at a temperature of 68.5°C for 20 hours. The log-log graph between the concentrations of WPI and *G*′ showed that the value of slope decreased with increasing NaCl concentration; therefore, the *D*_*f*_ value also decreased as NaCl concentration increased. This was in contrast to the observations done using permeability method, where the *D*_*f*_ value increased along with the increase of NaCl concentration. These statements differed from those of Ikeda et al. [20] and Vreeker et al. [8] who stated that the increase of NaCl concentration added in heat gelation method caused the decrease in *D*_*f*_ value. Despite the ambiguity, authors tried to extract the *D*_*f*_ value from the study. We observed that the gel belonged to the weak-link regime based on Shih et al. [63] with *D*_*f*_ 2.81 for the gel with addition of 0.1 M NaCl and 2.78 for 0.5 M NaCl. The result was in line with microscopy method (*D*_*f*_ = 2.20). Ikeda et al. [20] also examined WPI gel made by heat gelation method. The WPI solution was initially stirred for 1 hour in 25–1000 mM NaCl, adjusted to pH 7.0, diluted to the desired protein concentration, then heated from 25 to 90°C at a rate of 2.5°C/minute, and held for 1 hour. Gel produced based on Shih et al. [63] and strain as rheological parameter belonged to the strong-link regime, while, based on stress and storage modulus, the gel under this study was included in the weak-link regime. The *D*_*f*_ values determined in this study were based on the strong-link regime with *D*_*f*_ 2.2 (25 mM NaCl), 1.5 (100 mM NaCl), and 1.8 (500 mM NaCl). The difference of regime classification on some rheological parameters was then clarified by Wu and Morbidelli [10] who proposed that the gels were on the transition regime with *D*_*f*_ values of 2.56 (25 mM NaCl), 2.22 (100 mM NaCl), and 2.20 (500 mM NaCl). The results made more sense because the increase of NaCl concentration should accelerate the aggregation process, causing a decrease in *D*_*f*_ value [8]. This was also supported by research done by Foegeding et al. [92] which showed that microstructure image of gel at 100 mM NaCl comprised a mixture of gel with a fine-stranded and particulate morphologies, therefore indicating that, at 100 mM NaCl, a transition of microstructural changes of gel occurred. Verheul and Roefs [93] also showed that WPI gel formed with NaCl concentration of 0.4–3.0 M resulted in *D*_*f*_ value of 2.20 by using permeability method; nevertheless the fractal concept cannot simply be applied to WPI gels. The type of aggregation on heat gelation by using rheology method in general followed the reaction controlled aggregation with some limitations as already explained above. The resulting aggregation type was similar to microscopy method and even other methods.

Marangoni et al. [38] and Kuhn et al. [46] also conducted a comparison of *D*_*f*_ values resulting from the microscopy and rheology method in salt-induced cold gelation system. The resulting *D*_*f*_ values were 2.45 and 2.62 for the NaCl-induced gels and 2.63 and 2.66 for the CaCl_2_-induced gels. It was also observable that the gel produced by salt-induced cold gelation of whey protein followed the weak-link regime, although both researchers used different scaling models and rheological parameters to determine the regime and *D*_*f*_ values of gels. The *D*_*f*_ values generated from the salt-induced cold gelation method lied between 2.62 and 2.66; the results were similar to those observed using the microscopy method.

Vreeker et al. [8] examined acid-induced cold-set gel prepared from WPI solution with 1 and 10% protein (w/w) at pH 6.7 which was then heated at 70 and 90°C for 60 minutes. The solution was then cooled to 20°C and gel formation was induced by using 0.1 M HCl to a pH of 5.4. The results showed that the gel belonged to the weak-link regime with *D*_*f*_ value 2.0–2.5. Alting et al. [32] also examined the WPI gel by same gelation method, but using a WPI concentration of 9% (w/w) which was heated at 68.5°C and diluted to 0.5–9% (w/w). The solution was then stored at 40°C and GDL was added to a pH of 5. *D*_*f*_ values from various methods and scaling models were not obtained; this might be because gel made by acid-induced cold gelation process at a certain level (>100 nm) did not generate fractal structure, although the aggregate image generated from CSLM could give a *D*_*f*_ value of 2.2 on the same gel system. Andoyo et al. [34] observed the *D*_*f*_ value by using rheology method on the same acid-induced cold gelation system with observation by microscopy method. The scaling model used was that of Shih et al. [63] with storage modulus and limit of linearity as rheological parameters. Both rheological parameters produced similar *D*_*f*_ values, although for WPA system, *D*_*f*_ values from rheology method (1.15–1.7) were slightly different compared to the microscopy method (2.15). WPA gel belonged to strong-link regime and gel made from mixture of WPA and NMC belonged to weak-link regime with *D*_*f*_ value 2.29–2.6. The values were not of much difference with microscopy method (*D*_*f*_ 2.64–2.69). Eissa and Khan [90] also examined acid-induced cold-set gels made from WPI with or without enzyme modification. The results showed that the *D*_*f*_ value obtained from rheology (2.05–2.09 for strong-link regime) did not vary much with the microscopy method (1.96–1.98). Based on log-log graph from limit of linearity, the resulting gel belonged to strong-link regime, but based on log-log graph of elastic modulus, the resulting gel belonged to weak-link regime. Authors attempted to obtain *D*_*f*_ values of gels based on weak-link regime; results obtained were relatively larger (*D*_*f*_ 2.77–2.78). The observations also showed that microstructure and *D*_*f*_ values for both gels were similar, but the fracture stress and strain values were relatively different; this might be because several factors that affect the fracture properties of gel were not observable at microstructure level. The rate of aggregation resulting from acid-induced cold gelation method by using rheology method in general was varied among different researchers, ranging from 1.15 to 2.85. However, we could conclude that the gelation process was not ideal RLCA. The pretreatment of whey proteins can be done to modify the aggregation process so as to alter the *D*_*f*_ value, thus changing the type of aggregation [34, 94]. Detailed data presented above was collected in Table 3.

The values of *D*_*f*_ obtained from rheology method from most of the studies were not of much difference with the values of *D*_*f*_ obtained from microscopy, or even other, methods. This indicated that the various rheological properties of gel aggregates were reflection of the fractal structure of gel aggregate [34, 38, 46, 60, 68]. The salt-induced cold gelation process generally resulted in weak-link gels [38, 46, 51], whereas acid-induced cold gelation generally produced strong-link gel when the initial pH of protein solution was above 7.0 and weak-link gel when pH is below 7.0 [8, 32, 34, 51, 90, 95]. The strong-link gels were generally transparent (fine-stranded; gel formed at low ionic strength and pH far from pI), while weak-link gels were turbid (particulate; gel formed at high ionic strength and pH close to pI). Hagiwara et al. [60] therefore suggested that the transparency and turbidity of gel aggregates were universal characteristics for gels with both strong- and weak-link regime, respectively. Aggregation for all three types of gelation was reaction limited, but addition of other components or pretreatment of the protein prior to processing could be done to modify the aggregation process so as to produce structure, *D*_*f*_ values, regime classification, and different aggregation types.

From the above description regarding *D*_*f*_ value by microscopy and rheology, in general there were a variation of *D*_*f*_ values among researchers and differences of *D*_*f*_ values within the same gel system. These may occur due to several reasons; for example, different length scale used among researcher means that different resolution of the measurements and dynamic changes inside the gel make the scaling laws not able to be applied completely as explained by several authors. The fractal gel model assumes that the gel consists of crosslinked flocs that fill up the space. The flocs are supposed to be fractal structures formed by aggregated particles. The model allows for the possibility that different types of bonds are formed between particles within the flocs and between particles of different flocs. One way to obtain such a structure is by random aggregation of particles, which leads to fractal aggregates (flocs) that grow until they fill up the space and interconnect into a space filling network. Furthermore, the volume fraction of the particles used by authors was small so that the fractal character of the flocs can be expressed before they fill up the space. Different thresholding methods used for *D*_*f*_ values by microscopy lead to varied values of *D*_*f*_. This is one of the critical steps in using digital imaging for fractal analysis and different thresholding value can lead to different fractal dimensions. Thresholding is not straightforward and changing the threshold value can slightly change the fractal dimension. This is probably the reason why fractal dimensions calculated using different rheological and microscopy parameter are varied. However, the results are still on the same correspondence aggregation regime.

## 5. Conclusion

Results confirmed the applicability of fractal analysis by macroscopic or microscopic methods in describing whey protein-based gels, in which viscoelastic measurements correlated well with the microstructure of gels. This can be shown by the values of *D*_*f*_ obtained from rheological measurement which agreed to some extent with those from image analysis, an indication that the rheological behavior of the aggregate gels is a reflection of fractal structure of the aggregates in gels. Both methods tended to produce *D*_*f*_ values with same aggregation types for similar gel systems and even yielded *D*_*f*_ values similar to other methods. Results from numerous studies confirmed that fractal analysis from macroscopic and microscopic methods were applicable to quantify whey protein-based gels despite the presence of several limitations in both methods.

## Figures and Tables

**Table 1:** Scaling models for determining fractal dimension.

*A*	*B*	References	Gel classification
2/(3 − *D*_*f*_)		Bremer [85]	*(Straight strands)*

3/(3 − *D*_*f*_)	1/(*D*_*f*_ − 3)	Bremer [85]	*(Curved strands)*

(3 + *x*)/(3 − *D*_*f*_)	−1(1 + *x*)/(3 − *D*_*f*_)	Shih et al. [63]	*(Strong-link regime)*

1/(3 − *D*_*f*_)	1/(*D*_*f*_ − 3)	Shih et al. [63]	*(Weak*-*link regime)*

*β*/(3 − *D*_*f*_)	(3 − *β* − 1)/(3 − *D*_*f*_)	Wu & Morbidelli [10]	*β* = 1 + (2 + *x*)(1–*α*)
			*α* = 0 → *strong-link regime* *α* = 1 → *weak-link regime* 0 < *α* < 1 → *transition regime*

(Adapted from Alting et al. [32] and Wu and Morbidelli [10]).

**Table 2:** Fractal dimension (*D*_*f*_) values of different types of gels, measured by using microscopic method.

Reference	Gel type	*D*_*f*_
de Kruif et al. (1995)	*Salt*-*induced cold gelationβ*-Lg [NaCl] = 0.1 or 0.5 M	2.2

Hagiwara et al. (1997a)	*Heat*-*inducedβ*-Lg, pH = 7.0, [CaCl_2_] = 30 mM	2.7

Hagiwara et al. (1997b)	*Heat inducedβ*-Lg, pH = 7.0, [NaCl] = 1.0 M	2.68

Hagiwara et al. (1998)	*Heat*-*induced *BSA, I = 50 mM buffer pH 5.1, 0.1 M NaCl, II = 50 mM buffer pH 7.0, 30 mM CaCl_2_, III = 50 mM buffer pH 7.0, 5 mM CaCl_2_	I = 2.81 II = 2.81 III = 2.68

Marangoni et al. (2000)	*Heat*-*induced* WPI (5% w/v); *salt*-*induced cold gelation*	~2.45 for 300 mM NaCl at different protein concentration

Alting et al. (2003)	*Acid*-*induced cold gelation* WPI 9%	2.3

Kuhn et al. (2010)	*Heat*-*induced* WPI + 150 mM NaCl or CaCl_2_	CaCl_2_ = 2.82 NaCl = 2.81

Torres et al. (2012)	Yogurt with substituted microparticulate whey protein (MWP)	1.4–2.6

Andoyo et al. (2015)	*Acid*-*induced cold gelation* whey protein aggregates (WPA) & WPA + native micellar casein (NMC), pH 4.5	CSLM: WPA = 2.15 WPA + NMC = 2.64 TEM: WPA + NMC = 2.69

Eissa, Khan (2005)	Whey protein solution: 3% and 7.5% (heat with/without transglutaminase, TG), *low pH cold-set whey protein gel, final pH 4.0*	CSLM: 1.96–1.98 Rheology: 2.05–2.09

**Table 3:** Fractal dimension (*D*_*f*_) values of different types of gels, measured by using rheological method.

Reference	Gel type	*D*_*f*_
Hagiwara et al. (1997a)	*Heat induced*: BSA I = pH 7.0 II = 50 mM buffer pH 7.0, 30 mM CaCl_2_ III = 50 mM buffer pH 7.0, 5 mM CaCl_2_ *β*-Lg IV = pH 7.0 V = pH 7.0 + 30 mM CaCl_2_	I = strong-link, 2.00–2.07 II = weak-link, 2.82 III = weak-link, 2.61 IV = strong-link, 2.14–2.20 V = weak-link, 2.69

Hagiwara et al. (1997b)	*Heat induced β*-Lg pH = 7.0 [NaCl] = 1.0 M	Weak-link, 2.62

Hagiwara et al. (1998)	*Heat*-*induced *BSA I = 50 mM buffer pH 5.1, 0.1 M NaCl II = 50 mM buffer pH 7.0, 30 mM CaCl_2_ III = 50 mM buffer pH 7.0, 5 mM CaCl_2_	I = weak-link, 2.82 II = weak-link, 2.82 III = weak-link, 2.61

Vreeker et al. (1992)	*Acid*-*induced cold gelation* WPI (1 or 10% w/w) + 0.1 M NaCl or CaCl_2_ at pH 5.4	*G*′ Shih et al. (1990) = 2.0 Bremer (1992) = 2.3 *σ*: Bremer (1992) = 2.4–2.5

Ikeda et al. (1999)	*Heat*-*induced *WPI pH 7.0 + 25–1000 mM NaCl	25 mM NaCl = 2.2 100 mM NaCl = 1.5 *(incorrectly predicted)* 500 mM NaCl = 1.8 50, 80, 500, and 1000 mM NaCl = DLCA

Alting et al. (2003)	*Acid*-*induced cold gelation* WPI 9%	*D*_*f*_ 2.3, acid-induced cold gelation probably starts off as a fractal process but is rapidly taken over by another mechanism at larger length scales (>100 nm)

de Kruif et al. (1995)	*Salt*-*induced cold gelationβ*-Lg [NaCl] = 0.1 or 0.5 M	After passing the gelation threshold, gel with more or less fractal-like structure was formed and it coarsens with increasing salt concentration. Nevertheless, gels properties could not completely be described using the scaling laws as explained by many authors

Marangoni et al. (2000)	*Heat*-*induced* WPI (5% w/v) *Salt*-*induced cold gelation*	CaCl_2_ = weak-link, 2.63 NaCl = weak-link, 2.45

Stading et al. (1993)	*Heat*-*induced β*-Lg at pH 5.3 with different heating rate	Microstructure of gels showed straight-strand type pH 5.3 = 2.46–2.47 pH 5.7 = 2.91–2.94

Kuhn et al. (2010)	*Heat*-*induced* WPI + 150 mM NaCl or CaCl_2_	CaCl_2_ = weak-link, 2.66 NaCl = weak-link, 2.62

Andoyo et al. (2015)	*Acid*-*induced cold gelation* WPA & WPA + NMC, pH 4.5	WPA gels: strong-link *G*′ = 1.6–1.7 *γ*_0_ = 1.15 WPA + NMC gels: weak-link *G*′ = 2.6 *γ*_0_ = 2.29

Hagiwara et al. (1996)	BSA dissolved in HEPES buffer of pH 7.0 and acetate buffer of pH 5.1 to 0.1% and 0.001% solutions, heated at 95°C, varying the heating time	BSA at pH 7.0 were about 2.1 and 1.5; *D*_*f*_ of heat-induced aggregates at pH 5.1 was about 1.8

Foegeding et al. (1995)	A fine-stranded matrix formed in protein suspensions contained monovalent cation (Li^+^, K^+^, Rb^+^, and Cs^+^) chlorides, sodium sulfate, or sodium phosphate at ionic strengths ≤ 0.1 mol/dm^3^. This matrix varies in stress and strain at fracture at different salt concentrations	Protein-specific factors can affect the dispersibility of proteins and thereby determine the microstructure and fracture properties of globular protein gels

Verheul & Roefs (1998)	Gels were made at near-neutral pH. Protein concentration (35–89 g/l) and NaCl concentration (0.1–3 mol/dm^3^) were systematically varied	Gel structure did not change much after gel formation, while gel rigidity continued to increase, and at the gel point only part of the protein in the dispersion contributes to the gel network. The fractal concept cannot simply be applied to WPI gels

Eissa, Khan (2005)	Whey protein solution: 3% and 7.5% (heat with/without transglutaminase, TG), *low pH cold-set whey protein gel, final pH 4.0*	CSLM: 1.96–1.98 Rheology: 2.05–2.09
